# Iodized Salt May Not Be Sufficient to Guarantee an Adequate Iodine Intake in Pregnant Women

**DOI:** 10.3390/nu15194182

**Published:** 2023-09-27

**Authors:** Vincenzo Spina, Enke Baldini, Silvia Cardarelli, Cosimo Oliva, Stefano Venarubea, Franca Faraoni, Giovanni Pastore, Rachele Tittoni, Angela Musella, Antonia Squarcella, Eleonora Lori, Elisabetta Renzi, Roberta Feroci, Flavia Mastrodonato, Fabiola Ciferri, Camilla Virili, Marco Centanni, Cristina Fabiani, Rocco Rago, Michele Carlo Schiavi, Pierluigi Palazzetti, Eleonora D’Armiento, Vito Cantisani, Salvatore Sorrenti, Salvatore Ulisse

**Affiliations:** 1 Mother and Infant Department Unit, ASL-Rieti, 02100 Rieti, Italy; v.spina@asl.rieti.it (V.S.); e.renzi@asl.rieti.it (E.R.); r.feroci@asl.rieti.it (R.F.); f.mastrodonato@asl.rieti.it (F.M.); f.ciferri@asl.rieti.it (F.C.); 2 Department of Surgery, “Sapienza” University of Rome, 00185 Rome, Italy; enke.baldini@uniroma1.it (E.B.); silvia.cardarelli@uniroma1.it (S.C.); eleonora.lori@uniroma1.it (E.L.); salvatore.sorrenti@uniroma1.it (S.S.); 3 Obstetrics and Gynaecology Unit, “S. Camillo De Lellis” Hospital, 02100 Rieti, Italy; c.oliva@asl.rieti.it (C.O.); g.pastore@asl.rieti.it (G.P.); rachelet@virgilio.it (R.T.); angela.musella@gmail.com (A.M.); a.squarcella@asl.rieti.it (A.S.); 4 Clinical Pathology Laboratory Unit, “S. Camillo De Lellis” Hospital, 02100 Rieti, Italy; s.venarubea@als.rieti.it; 5 Pediatrics and Neonatology Unit, “S. Camillo De Lellis” Hospital, 02100 Rieti, Italy; f.faraoni@asl.rieti.it; 6 Department of Medico-Surgical Sciences and Biotechnologies, “Sapienza” University of Rome, 04100 Latina, Italy; camilla.virili@uniroma1.it (C.V.); marco.centanni@uniroma1.it (M.C.); 7 Mother and Infant Department, “Sandro Pertini” Hospital, 00157 Rome, Italy; cristina.fabiani@aslroma2.it (C.F.); rocco.rago@aslroma2.it (R.R.); 8 Obstetrics and Gynaecology Unit, “Sandro Pertini” Hospital, 00157 Rome, Italy; michele.schiavi@aslroma2.it (M.C.S.); pierluigi.palazzetti@aslroma2.it (P.P.); 9 Department of Internal Medicine and and Medical Specialties, “Sapienza” University of Rome, 00185 Rome, Italy; eleonora.darmiento@uniroma1.it; 10 Department of Radiological and Oncological Sciences and Pathological Anatomy, “Sapienza” University of Rome, 00185 Rome, Italy; vito.cantisani@uniroma1.it; 11 Teleradiology Complex Unit, ASL-Rieti, 02100 Rieti, Italy

**Keywords:** iodine, pregnancy, thyroid volume, thyroid hormones, iodized salt, iodine-containing supplements

## Abstract

Adequate iodine intake is of crucial importance in pregnancy to meet the thyroid hormone needs of both mother and fetus. In the present study, undertaken as a part of the surveillance actions following the introduction in Italy of a national salt iodination program in 2005, the iodine intake was investigated in 123 pregnant women and 49 control women living in the same area of central Italy. All the participants were screened for urinary iodine concentration (UIC), serum level of thyrotropin, free-thyroxine, free-triiodothyronine, and thyroid volume. Moreover, they were provided with a questionnaire on the use of iodine-containing salt or supplements. Control women had a median UIC of 102 µg/L, consistent with an iodine sufficiency, while in pregnant women the median UIC value was 108 µg/L, lower than the endorsed UIC of 150 µg/L. In addition, pregnant women showed a significantly increased median thyroid volume compared to controls. Interestingly, the median UIC did not differ between pregnant women not using iodine-containing salt or supplements and those regularly consuming iodized salt alone, while pregnant women with a daily intake of iodine-containing supplements had an adequate median UIC (168 µg/L). In conclusion, the data reported here showed that pregnant women and their fetuses are still exposed to the detrimental effects of iodine deficiency and that the consumption of iodine-containing supplements should be recommended in pregnancy.

## 1. Introduction

Iodine is a micronutrient endowed with several physiological functions [1], the main one being its role in the biosynthesis of the thyroid hormones (THs) T_4_ (3,5,3′,5′-tetraiodo-L-thyronine) and T_3_ (3,5,3′-triiodo-L-thyronine) in the thyroid gland [1]. The daily iodine intake recommended by the World Health Organization (WHO), the United Nations International Children’s Emergency Fund (UNICEF), and the Iodine Global Network (IGN) in adults is 150 µg [2,3,4,5]. This dose should be augmented during gestation to 200–300 µg/day to support the changes occurring in pregnancy, that is a faster peripheral T_4_ metabolism, onset of fetal thyroid function, higher urinary iodide excretion, and augmented maternal T_4_ requirement resulting from the estrogen (E2)-induced increase in circulating thyroxine-binding globulin (TBG) [2,3,4,5]. In particular, during the first trimester of gestation, an adequate maternal T_4_ plasma level is of primary importance since, following placental transfer, it represents the main source of T_4_ for the fetus [1,2,3]. The daily iodine consumption should continue to be elevated during breastfeeding (250–290 µg/day) to warrant the optimal concentration (roughly 115–150 µg/day) of iodine in the milk [6].

Iodine deficiency has several detrimental consequences on human health throughout the lifetime, acknowledged as iodine deficiency disorders (IDD) [7,8]. During pregnancy, a poor iodine intake has been shown to cause harmful effects on the fetus, comprising delayed development and brain maturation. In fact, suitable THs levels are necessary for neural migration and brain myelinization during the fetal and postnatal period [9,10,11,12,13]. Depending on the level of maternal iodine deficiency, a continuum of alterations ranging from mild mental retardation to severe neurological impairment can occur, whereas infantile cretinism results from a severe iodine deficiency during pregnancy and occurs more frequently in geographical areas with a prevalence of goiter >5% [9,10,11,12,13]. In addition, iodine deficiency in pregnancy leads to maternal and fetal goiter, may induce miscarriages, stillbirths, delayed fetal growth, neonatal hypothyroidisms, and reduced fertility in adults [1]. It is worthwhile to mention that also in areas characterized by an adequate iodide intake a substantial number of pregnant women have UIC values below the normal range [14,15,16,17]. This evidence prompted the Public Health Committee of the American Thyroid Association to recommend a comprehensive iodine supplementation for pregnant women in the United States and Canada [18]. In the present study, undertaken as a part of the surveillance program implemented by the Italian National Observatory for Monitoring Iodine Prophylaxis (OSNAMI) following the introduction in Italy of a national salt iodination program (30 mg/kg) in 2005, we show that most pregnant women of Rieti, a city in the Lazio region of central Italy, are still exposed to the damaging effects of iodine deficiency despite an adequate iodine intake of control non-pregnant women.

## 2. Materials and Methods

### 2.1. Subjects

From April 2021 to March 2023, 49 clinically healthy non-pregnant women of childbearing age and 124 pregnant women (47 at the first trimester, 37 at the second trimester, and 40 at the third trimester) were enrolled on presentation at the Mother and Infant Department or at the Obstetrics and Gynaecology Unit of the “S. Camillo De Lellis” hospital of Rieti. All participants provided informed consent and the study protocol was approved by the Ethical Committee Lazio 1 of the Regional Health System (Protocol n. 288/CELazio1). Only women without current or previous thyroid diseases and pregnant women with a normal gestation were included. All of them were resident in the urban area of Rieti of central Italy.

Women were subjected to ultrasound examination of the thyroid gland, had a blood draw, and provided a morning urine sample, which was frozen to −20 °C until assayed. Moreover, they were asked to fill in a written questionnaire on their nutritional lifestyles. More precisely, it was inquired whether they regularly use iodized salt and/or vitamins/mineral supplements containing iodine. The questionnaire contained also questions about educational level, number of previous pregnancies and eventual abortions, type of diet followed (i.e., varied, vegetarian, or vegan), how much water was drunk daily, smoking habits, if they regularly used mouthwashes containing iodine, or if they recently used iodine-based disinfectants. Some women, however, did not provide their blood or morning urine samples or did not perform thyroid ultrasonography. Therefore, the precise number of women evaluated is indicated in each figure of the Result section.

### 2.2. Urinary Iodine Concentration (UIC) Determination

The UIC of morning urine specimens was measured using the Sandell–Kolthoff method using a commercial colorimetric kit (Cell-tech, Turin, Italy), and reported in µg/L. The intra- and inter-assay coefficients of variation (CVs) were, respectively, 8.6% and 11.9%.

### 2.3. TSH, FT_4_, FT_3_, and Thyroid Auto-Antibody Assays

Serum levels of anti-Tg and anti-TPO autoantibodies, TSH, FT_4_, and FT_3_ were determined by immunoassay in the cobas^®^ 6000 analyzer (Roche Diagnostics, Mannheim, Germany). Reference ranges were as follows: Anti-Tg < 20 UI/mL; Anti-TPO < 10 UI/mL; TSH 0.35–4.9 µUI/mL; FT_4_ 0.7–1.48 ng/dL; and FT_3_ 2.0–4.4 pg/mL.

### 2.4. Thyroid Ultrasound

The ultrasonographer Samsung WS80A (Suwon, Republic of Korea) equipped with a linear transducer was employed to perform thyroid ultrasonography. Longitudinal (Ld), transverse (Td), and anteroposterior (APd) diameters of each thyroid lobe were recorded to calculate thyroid volume (TV) through the following formula applied to each lobe:

TV (mL) = Ld × Td × APd × π/6.

The same observer (VS) performed all the ultrasound examinations. The intra-observer variability, calculated as between-visit CV, was evaluated using six measurements made on six different subjects and ranged from 4.9% to 6.8%.

### 2.5. Statistical Analysis

At first, the Shapiro–Wilk test for normality was applied to all continuous variables and none of them, except FT_3_, were found to follow a normal distribution. Therefore, the non-parametric Mann–Whitney and Kruskal–Wallis tests, with the Bonferroni post hoc test, were applied to assess differences between two groups or more than two groups, respectively. All tests were run using the IBM SPSS Statistics, version 27 (Armonk, NY, USA). Values were considered statistically significant when the pertaining *p* value was <0.05.

## 3. Results

**Figure 1:**
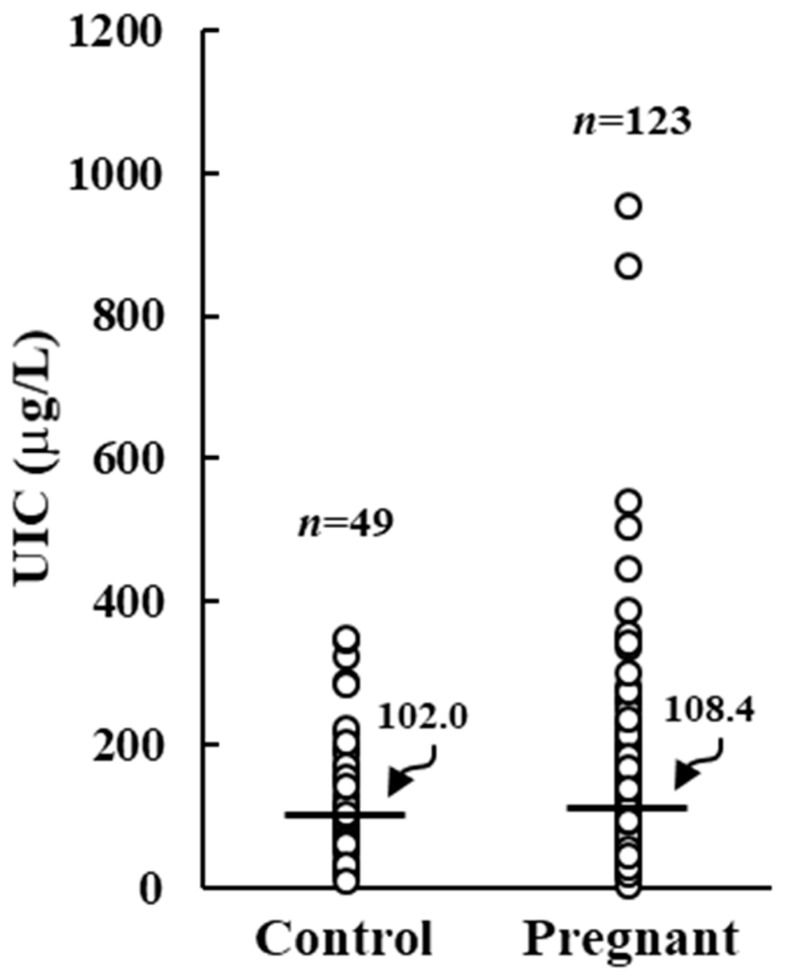
Urinary iodine concentration (UIC) in control and pregnant women. Horizontal bars indicate the median values.

One hundred twenty-four pregnant women with a median age of 33 year (range 18–45 year) and 49 control women with a median age of 37 year (range 20–48 year) were analyzed. Control women showed a median UIC of 102.0 µg/L (range 9.8 µg/L to 348.8 µg/L), suggesting an adequate iodine intake. On the other hand, pregnant women had a median UIC value of 108.4 µg/L (ranging from 3.9 µg/L to 654.3 µg/L), not statistically different from control group but lower than the threshold value of 150 µg/L (Figure 1).

Specifically, five (4.1%) pregnant women had an UIC below 20 µg/L, nineteen (15.5%) had an UIC between 20 and 49 µg/L, and fifty-three (43.1%) had an UIC between 50 and 149 µg/L. An adequate iodine intake was recorded in 35 (28.5%) pregnant women, with an UIC between 150 and 299 µg/L. Finally, 11 (8.9%) showed an excessive UIC ≥ 300 µg/L.

**Figure 2:**
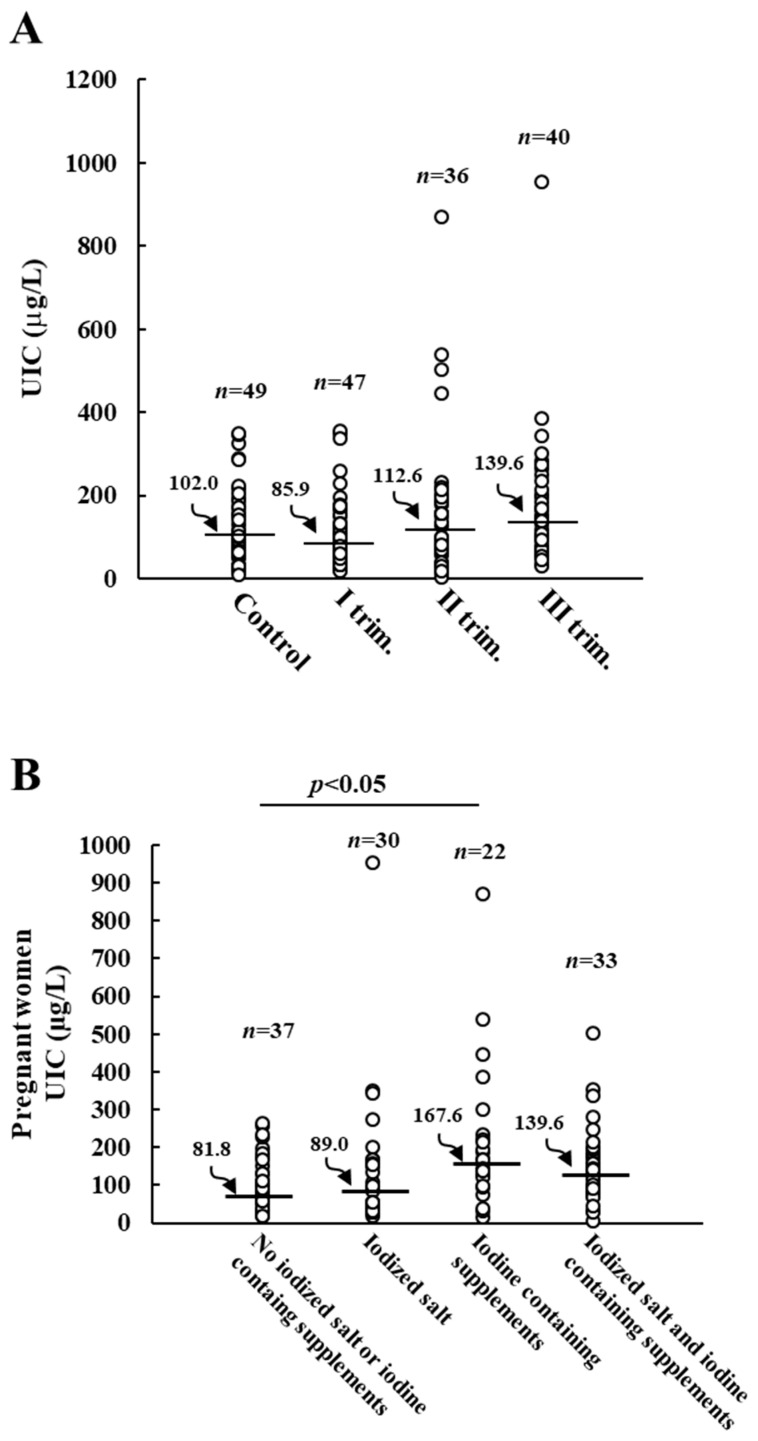
Urinary iodine concentration (UIC) in control and pregnant women. Panel (**A**): UIC of control and pregnant women grouped by trimester of pregnancy. Panel (**B**): effects of regular consumption of iodine-containing salt and/or supplements on UIC values of pregnant women. Horizontal bars indicate the median values.

When pregnant women were grouped based on the gestation time, the lowest UIC median value (85.9 µg/L) was noticed in those at the first trimester of pregnancy (Figure 2A). The median UIC tended to be higher in the second and third trimester but remained below the recommended value (Figure 2A). The consumption of iodized salt and/or iodine-containing supplements during these months was checked. Iodine-containing supplements were taken by 9 out of 47 pregnant women in the first trimester, by 23 out of 36 women in the second trimester, and by 24 out of 40 women in the third trimester. Therefore, it is likely to assume that the observed increase in median UIC, although not statistically significant (*p* = 0.078), is due to those women who started taking daily supplements in the second and third trimester. As reported in Figure 2B, pregnant women not regularly using iodized salt or iodine-containing supplements showed a UIC median value of 81.8 µg/L, which was not significantly improved by the regular use of iodized salt alone. On the contrary, pregnant women with a daily intake of iodine-containing supplements had an adequate iodine intake (median UIC 167.6 µg/L). Also, pregnant women regularly using iodized salt and iodine-containing supplements had an increased median UIC value (139.6 µg/L) (*p* = 0.052) compared to women not using either iodized salt or iodine-containing supplements.

**Figure 3:**
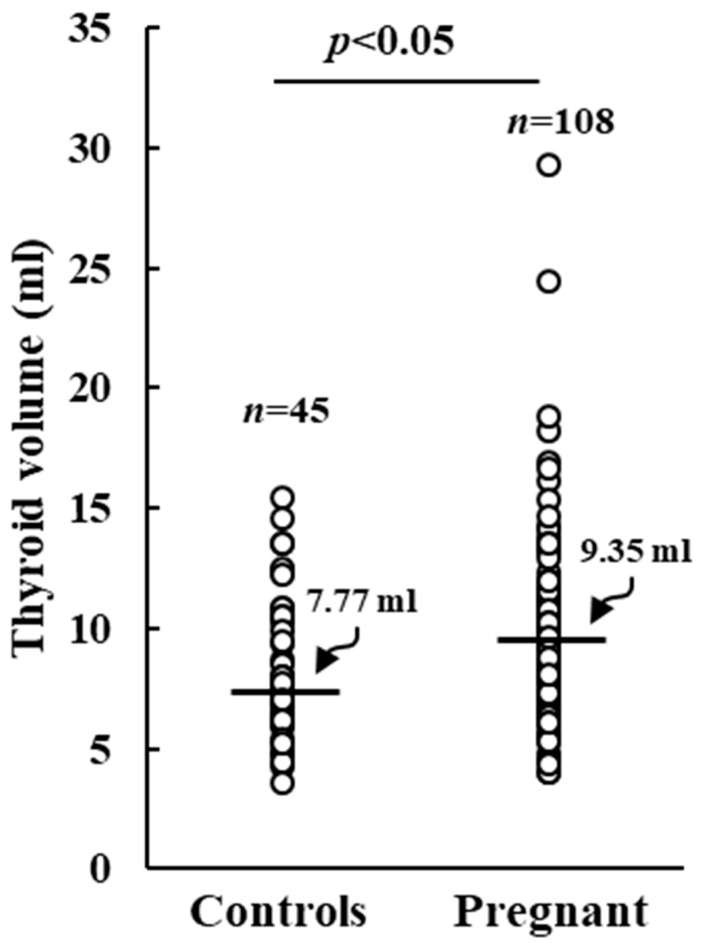
Thyroid volume in control and pregnant women. Horizontal bars indicate the median values.

No correlation could be observed between UIC and thyroid volume, TSH, FT_3_, or FT_4_ levels was observed. Pregnant women displayed a significantly augmented median thyroid volume (TV) compared to control ones (9.35 mL vs. 7.77 mL, *p* < 0.05), as shown in Figure 3.

**Figure 4:**
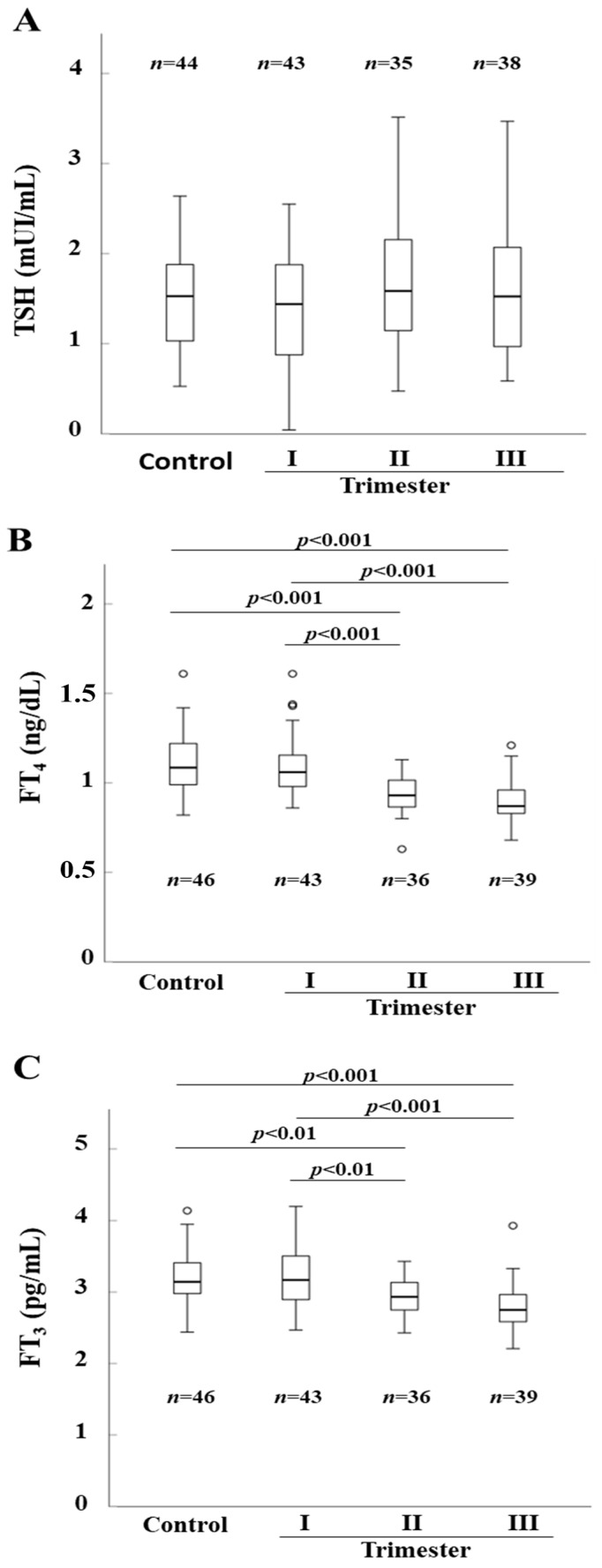
Thyroid stimulating hormone (TSH) (Panel (**A**)), FT_4_ (Panel (**B**)), and FT_3_ (Panel (**C**)) serum levels in control and pregnant women. Horizontal bars indicate the median values.

Serum levels of TSH, FT_4,_ and FT_3_ in control and pregnant women are reported in Figure 4. As shown in Figure 4A, TSH levels did not change significantly between control and pregnant women at the different trimesters of gestation. On the other hand, serum levels in both FT_3_ and FT_4_ were significantly lower in the pregnant women in the second and third trimester compared to those at the first trimester and to controls (Figure 4B,C).

## 4. Discussion

In 2020, the Iodine Global Network (IGN) scorecard reported Italy among the iodine sufficient countries based on median UIC in school age children (SAC), for the first time [19,20,21]. This accomplishment is a consequence of the nationwide program of iodine prophylaxis promulgated in 2005 (law n.55/2005) and of effective monitoring performed by the OSNAMI [20,21]. In particular, the law expects that all stores ensure the concomitant availability of iodine-enriched salt (30 mg/kg) and common salt, with the latter available only upon explicit request of the consumer. It also recommends the use of iodized salt in the catering area. Nonetheless, there is still widespread consumption of non-iodized salt in the Italian population. Several factors contribute to this trend, and primarily the fact that the rules contained in the law n.55/2005 remain largely disregarded, with most local retailers selling both iodized and non-iodized salt or even, in a minority of cases, only non-iodized salt [22]. This, along with the lower cost of non-iodized salt and the lack of an effective information campaign and public awareness of the adverse effects of iodine deficiency may account for the still relative high consumption of non-iodized salt.

In the present study, undertaken as a part of the surveillance program endorsed by the aforementioned law, the iodine intake of control non-pregnant women and pregnant women at different trimesters of gestation was investigated. The data obtained showed that, while control women had an adequate median UIC, pregnant ones had a median UIC well below the recommended value. These findings confirm previous reports published in 2008 and in 2018, showing a low iodine intake in pregnant women in the same region of central Italy [17,23,24]. Therefore, our observations indicate that the current iodine prophylaxis program adopted in Italy may be sufficient to guarantee the required iodine intake in non-pregnant women but not in pregnant ones, which are still exposed, together with their fetuses, to detrimental consequences of iodine deficiency. Actually, it is known from previous studies that even in regions with adequate iodide intake a considerable number of pregnant women display an insufficient iodine intake due to the increased iodine demand during gestation [14,15,16,18,25].

It is also worth mentioning that the cut-offs for adequate UIC in control (100 µg/L) and pregnant women (150 µg/L) were introduced by the WHO considering a daily urinary volume of 1.5 L. However, the higher glomerular filtration rate occurring during pregnancy may cause an increased daily urinary volume, that may render the UIC cut-off of 150 µg/L unreliable. Indeed, a previous study executed on pregnant women of an iodine adequate area of China demonstrated that the UIC/creatinine ratio reflects the 24 h iodine excretion better than UIC measured on spot urine samples [26]. According to this assumption, in the present work iodine deficiency detected in pregnant women may be overvalued. However, previous studies demonstrated that in iodine repleted areas the thyroid volume should not change during pregnancy [27,28]. In our cohort, we observed a significant increase in the median thyroid volume in pregnant women compared to that of control women, which suggests a condition of iodine deficiency.

In a previous cross-sectional multicenter study, it has been reported that the use of iodized salt provides sufficient dietary intake to ensure adequate iodine nutrition in all populations, including pregnant and lactating women, and in breastfed infants only when the use of iodized salt covers a large proportion (≥90%) of the total amount of salt consumed [29,30]. In Italy, the latest survey performed in the period 2016–2019 indicated that about 71% of all salt consumed was iodine-enriched and that 78% of the school canteens used iodized salt [31]. Thus, it is possible that this still relatively low consumption of iodized salt, while sufficient in the general population, remains inappropriate in pregnant women. Among these, women not using iodized salt or iodine-containing supplements had a low median UIC (82 µg/L) which, however, did not significantly differ in pregnant women regularly using iodized salt (89 µg/L). On the other hand, pregnant women on iodine-containing supplements showed an adequate median UIC (168 µg/L). These observations suggest the opportunity of introducing the use of iodine supplementation as soon as pregnancy is started or even when pregnancy is planned, as recommended in China, the United States, and Canada by the Public Health Committee of the American Thyroid Association [18,32,33].

Finally, no significant changes could be observed in the median TSH serum level between control and pregnant women in the different trimesters of gestation, while FT_4_ and FT_3_ serum concentrations were significantly lower in pregnant women in the second and third trimester of gestation as compared to control and pregnant women at the first trimester of gestation. These data appear to corroborate previous studies documenting a physiological gradual decrease of FT_4_ serum levels with the increasing gestational age [34,35,36,37].

## 5. Conclusions

In conclusion, we showed that in an area of central Italy pregnant women and their fetuses are still unprotected from the detrimental consequences of iodine deficiency despite an ongoing national universal salt iodination program and that the regular use of iodized salt is not per se sufficient to cover the augmented demand for iodine intake in pregnancy. These observations, besides encouraging greater attention from major healthcare providers, including obstetricians and gynecologists, should recommend generalized consumption of iodine-containing supplements during pregnancy.

## Data Availability

All data are available upon request to the corresponding author.
